# Notes and News

**Published:** 1897-03-20

**Authors:** 


### Notes and News.

(Collected and Communicated.)

### HOSPITAL MEETINGS.

Royal Sea Bathing Infirmary, Margate.—General Court, on Monday, March 22nd, at 3.30 p.m., at the Offices, 30, Charing Cross.

The Middlesex Hospital has received a munificent gift of one thousand guineas, from Mrs. Sarah Moore, to endow a bed in the cancer wards, to be called the Moore Thompson bed.

The latest reports from Bombay show that the Plague Committee are working with energy and system. The diminution of death from all causes argues an improvement, but it is still impossible to differentiate with accuracy between the deaths from the plague and from other causes. Upwards of 500 pilgrims intending to journey to Mecca from Bombay were placed under observation for ten days, and finally dispersed to their homes without giving any trouble.

In this time of distress in India, the sympathy of English people has been very keenly aroused, and all are eager to lend a helping hand, not only by gifts in money but in kind. In respect to this last, the public have been in the position of not knowing how to secure the proper distribution of their gifts. Many irresponsible persons have come forward with appeals, but however worthy in motive these persons may be, they have not satisfied cautious persons that in a country like India, where everything is worked through official sources, they will be able to guarantee the intended application of gifts entrusted to them. The Mansion House has placed itself in complete communication with Indian officials, and through this channel only the public will be wise to offer their gifts to the sick or starving natives of India. Therefore we may welcome the arrangements, sanctioned by the approval of the Lord Mayor, which have been made to send out clothing and gifts in kind to be distributed under the direction of the Famine Fund Committee through local agencies. All correspondence on the subject is undertaken by Mrs. Hauser (the honorary secretary), 48, Bedford Gardens, Kensington, W.

An international congress of leprologists is to be held at Berlin; the date, however, is not yet fixed. Most Governments have undertaken to send representatives.

Miss Fisher, a literary contributor to the *Sunshine* magazine, has handed over a collection of £500 to the Southport Infirmary to found a cot to be named "The *Sunshine* Cot." The interest of many children and older people, too, was aroused by a true story of a small hospital patient, told by Miss Fisher, and a subscription list was opened, which at the end of five years reaches the necessary sum for an endowment, and also for the purchase of the little cot itself.

It is proposed to erect an infectious hospital for Bromsgrove, and the question of site, as is usual on such occasions, is causing a great deal of disturbance in the neighbourhood. From what has transpired it appears not unlikely that accommodation for small-pox of a permanent or temporary nature is contemplated, and this being the case, strong objections to the only site suggested, at Hill Top, are being raised, and these are to be brought before the County Council of Worcestershire.

An incident occurred recently which has caused some friction between the doctors on the medical staff of the infirmary and the private practitioners of Chelmsford. It appears that a gentleman met with an accident, and was attended by one of the general practitioners of the town, who, in the interest of his patient, secured admission for him into the infirmary. As, however, the private wards at the infirmary were all occupied, temporary quarters were arranged for him in the operating-room. An operation was necessary, and was performed by the surgical staff of the infirmary. The practitioners of Chelmsford addressed a letter to the managers of the infirmary, asking that in future, when a patient was admitted under similar circumstances, that he should be attended by his own medical attendant. The committee replied that they could not alter their rules.

We are pleased to see that the Adelaide Children's Hospital has made such steady progress since its establishment just two decades ago. During the last 10 years the accommodation has been nearly doubled, and that its usefulness is not to remain at a standstill is evident, seeing that, the necessity for isolation wards being admitted, generous friends, the Hon. J. and Mrs. Duncan, came forward, and contributed £1,250 on the condition that a like sum should be provided by others. This was shortly done, and a further sum, equalling £2,500, still being required, subscriptions have been received, leaving only £700 to be collected before the buildings are completed. It is not the sick of Adelaide only who will benefit by the extension of the hospital, but an advantage will accrue to the community at large through the destruction of a number of small cottages, whose inhabitants being undesirable rendered the neighbourhood less healthy and peaceful than it need have been. We may mention one more improvement at the hospital, namely, the lengthened stay now permitted to children suffering from diphtheria, which renders less probability of a spread of the disease by patients leaving the institution.

